# Absence of Effects of L-Arginine and L-Citrulline on Inflammatory Biomarkers and Oxidative Stress in Response to Physical Exercise: A Systematic Review with Meta-Analysis

**DOI:** 10.3390/nu15081995

**Published:** 2023-04-21

**Authors:** Andrey A. Porto, Luana A. Gonzaga, Cicero Jonas R. Benjamim, Vitor E. Valenti

**Affiliations:** 1 São Paulo State University (UNESP), Presidente Prudente 19060-080, SP, Brazil; 2 Autonomic Nervous System Center, São Paulo State University (UNESP), Marília 17525-900, SP, Brazil; 3 Department of Internal Medicine, Ribeirão Preto Medical School, University of São Paulo, Ribeirão Preto 14049-900, SP, Brazil

**Keywords:** L-Citrulline, L-Arginine, nitric oxide, defense system, oxidative stress, physical exercise, immunology, nutritional compounds, exercise, systematic review

## Abstract

Background: The repercussions on oxidative and inflammatory stress markers under the effects of arginine and citrulline in response to exercise are not fully reached. We completed a systematic review to investigate the effects of L-Citrulline or L-Arginine on oxidative stress and inflammatory biomarkers following exercise. EMBASE, MEDLINE (PubMed), Cochrane Library, CINAHL, LILACS, and Web of Science databases were used to record the trials. This study includes randomized controlled trials (RCTs) and non-RCTs with subjects over 18 years old. Those under the intervention protocol consumed L-Citrulline or L-Arginine, and the controls ingested placebo. We recognized 1080 studies, but only 7 were included (7 studies in meta-analysis). We observed no difference between pre- vs. post-exercise for oxidative stress (subtotal = −0.21 [CI: −0.56, 0.14], *p* = 0.24, and heterogeneity = 0%. In the sub-group “L-Arginine” we found a subtotal = −0.29 [−0.71, 0.12], *p* = 0.16, and heterogeneity = 0%. For the “L-Citrulline” subgroup we observed a subtotal = 0.00 [−0.67, 0.67], *p* = 1.00, and heterogeneity was not applicable. No differences were observed between groups (*p* = 0.47), and I² = 0%) or in antioxidant activity (subtotal = −0.28 [−1.65, 1.08], *p* = 0.68, and heterogeneity = 0%). In the “L-Arginine” sub-group, we found a subtotal = −3.90 [−14.18, 6.38], *p* = 0.46, and heterogeneity was not applicable. For the “L-Citrulline” subgroup, we reported a subtotal = −0.22 [−1.60, 1.16], *p* = 0.75, and heterogeneity was not applicable. No differences were observed between groups (*p* = 0.49), and I² = 0%), inflammatory markers (subtotal = 8.38 [−0.02, 16.78], *p* = 0.05, and heterogeneity = 93%. Tests for subgroup differences were not applicable, and anti-inflammatory markers (subtotal = −0.38 [−1.15, 0.39], *p* = 0.34 and heterogeneity = 15%; testing for subgroup differences was not applicable). In conclusion, our systematic review and meta-analysis found that L-Citrulline and L-Arginine did not influence inflammatory biomarkers and oxidative stress after exercise.

## 1. Introduction

Physiological stress situations, such as physical exercise, lead to oxidative stress associated with free radicals and reactive oxygen species (ROS) generation [1]. An imbalance between pro-oxidant generation and the physiological capacity to scavenge free radicals and ROS increases levels of oxidative stress [2]. During physical exercise, myokines are created and released by muscle fibers such interleukin-6 (IL-6), which is a pro-inflammatory myokine. Other myokines such as interleukin-10 (IL-10) and tumor necrosis factor-alpha (TNF-alpha) are also expressed during inflammatory conditions [3].

In this line, L-Citrulline is a non-essential amino acid well known as the precursor of L-Arginine in the urea cycle. L-Citrulline action via kidneys, tissues, and other vascular endothelial cells elevates plasma and tissue levels of L-Arginine. Consequently, L-Arginine is the substrate for the endothelial production of nitric oxide (NO), a potent vasodilator produced in the endothelium [4]. It is well known in the literature that the integrity and function of the endothelium, a dynamic autocrine and paracrine organ with vasodilator and anti-inflammatory properties, is crucial for executing such physiological functions [5]. In addition, there is a relationship between oxidative stress and inflammation, with each of these two processes influencing the other and creating a vicious circle capable of generating and maintaining an inflammatory process [6]. ROS and oxidative stress are essential to generate the physiological adaptations evoked by physical exercise [7], but in cases of excessive oxidative stress, recovery can be beneficiated by exogenous compounds to avoid physiological disturbances and improve recovery from strenuous exercise [8].

Limited evidence suggests that nutritional supplements containing antioxidant substances can have a positive influence on exercise performance through reduction in exercise-related oxidative stress, thus enhancing recovery [9]. Although these substances seem safe for human consumption and rarely present adverse effects [7,8,9], they may negatively influence training adaptations [10,11]. L-Arginine has been studied to decrease the release of pro-inflammatory myokines produced during an exercise session [12,13,14]. Therefore, the evaluation of dietary compounds with antioxidant and anti-inflammatory properties is an interesting line of research for the development of different nutritional interventions that may help immune system responses after physical exercise.

Some studies have explored the effects of L-Arginine and L-Citrulline intake on inflammatory and antioxidant responses to exercise. However, due to conflicting results and small sample sizes, there is no consensus on the results achieved among the primary studies. To date, effect estimators have not been performed to assess the repercussions on oxidative and inflammatory stress markers under the effects of L-Arginine and L-Citrulline compared to placebo after exercise. Understanding the results of the influence of these substances in the mentioned clinical parameters and health patterns is necessary to improve viable strategies to help athletic populations. In order to resolve these issues, we conducted a systematic review and applied a meta-analytic model to study the influence of L-Citrulline and L-Arginine on antioxidants, oxidative stress, and inflammatory and anti-inflammatory markers.

## 2. Materials and Methods

### 2.1. Registration

The review was reported according to the recommendations of the Preferred Reporting Items for Systematic Reviews and Meta-Analyses (PRISMA) [15] and is registered in the PROSPERO database (CRD42022359806).

### 2.2. Search Strategy and Study Selection

The searches were performed via EMBASE, MEDLINE/PubMed (via National Library of Medicine), Cochrane Library, CINAHL, Scopus, and Web of Science databases with the submission of the keywords “L-Arginine” OR “L Arginine” OR “Arginine” OR “DL-Arginine” OR “DL Arginine” OR “L-Citrulline” OR “Citrulline” AND “Interleukin-1” OR “Interleukin-4” OR “Interleukin-6” OR “Interleukin-10” OR “C-Reactive Protein” OR “Tumor Necrosis Factor-alpha” OR “Malondialdehyde” OR “Catalase” OR “Thiobarbituric acid reactive substances” OR “Superoxide dismutase” OR “Glutathione” OR “Inflammation mediators” OR “Antioxidants” OR “Ferric reducing ability of plasma” AND “Exercise” OR “Physical activity” OR “Sports “. No restrictions were applied to the studies’ languages or dialects (see search strategy in Supplementary Material).

All articles acknowledged were exported to the Rayyan QCRI program (Qatar Computing Research Institute, Doha, Qatar) to eliminate duplicates. The studies were screened in the Rayyan program by reading the title and abstract. The suitability stage was completed by two independent reviewers (AAP and LAG) reading the full articles. Another reviewer was invited to give a decision (VEV) if there was a disagreement concerning a study.

The studies needed to originate from peer-reviewed journals, published from the start of the database until 29 August 2022. For inclusion, the articles needed to achieve all the criteria described below: randomized control trial (RCT) or non-RCT design; participants older than 18 years; the intervention group consuming arginine or citrulline (powder or capsule); and the control group intaking placebo. L-Arginine and L-Citrulline combined with other compounds were not considered for this paper. The included studies analyzed the outcomes of oxidative stress and inflammatory markers measured before and post-exercise. Due to the limited amount of evidence on this topic, we did not delimitate a specific type of exercise, with all modalities eligible to be included (e.g., endurance, resistance exercise, submaximal aerobic exercise, and incremental testing) in this review.

### 2.3. Data Extraction

Data concerning the author, study design, features of the study participants, intervention, and exercise protocols of the respective studies were extracted from primary studies and presented in Table 1. Missing data were requested by contacting the corresponding study authors. This stage was finished independently by two reviewers (AAP and CJRB). When the author’s correspondent did not respond, the Web Plot Digitizer^®^(San Diego, CA, USA) was applied to extract the data presented in graphs. The post-intervention data were charted as mean and standard deviations (SD). Values presenting “standard error” or “confidence intervals” (CI) in the primary studies were then converted to SD.

### 2.4. Assessment of the Risk of Bias

The analysis of bias was executed using risk of bias tools by the Cochrane organization [20] via the Review Manager program (RevMan 5.4.1). Risk of bias is an instrument based on domains [21], and its evaluation is divided into seven fields: “Random sequence generation”, “Allocation concealment”, “Blinding of participants and personnel”, “Blinding of outcome assessment”, “Incomplete outcome data”, “Selective reporting”, and “Other Bias”. Classification is separated into three direct responses: low risk, unclear risk, and high risk. Our deductions were based on the table developed by Carvalho et al. [21], “Reviewers’ judgment and criteria for judgment”. Two independent authors completed the risk of bias analysis (AAP and LAG). Another (VEV) was consulted if there were any inconsistencies in their decisions.

### 2.5. GRADE (Levels of Evidence)

The Grades of Recommendation, Assessment, Development, and Evaluation (GRADE) Working Group (GRADE Working Group, McMaster University, Hamilton, ON, Canada, 2004) was enforced to examine the certainty of the evidence, as well as the study design of non-randomized (weak evidence) or randomized trials (strong evidence). Study quality (detailed study methods and execution) and significant limitations were considered secondarily in the strength of evidence analysis [22]. The summary of achievements was formed via GRADEpro GDT version 4^®^ (McMaster University, Hamilton, ON, Canada).

### 2.6. Qualitative Analysis (Systematic Review)

A narrative synthesis was implemented to describe detailed data on how each study was completed. The details for each study were introduced in texts and tables. The results of the individual qualitative analysis per study were completed by analyzing oxidative stress and inflammatory markers for the intervention or placebo protocols.

### 2.7. Quantitative Analysis (Meta-Analysis)

In the meta-analysis, we introduced oxidative stress, antioxidants, inflammatory and anti-inflammatory marker clinical values. The information required to construct the meta-analysis was the period post-intervention (after exercise). To assess oxidative stress outcomes, the indexes included were: thiobarbituric acid reactive substances (TBARS), carbonyls, uric acid (mg/dL), malondialdehyde (MDA) (μM), neutrophil MDA (mmol/106 cells), creatine (CK), and lactate dehydrogenase (LDH). Regarding the antioxidants outcomes, we included: superoxide dismutase (SOD) (U/mL), catalase (nmol/min/mL), and glutathione peroxidase (GPx). Inflammatory markers included were: interleukin-6 (IL-6) and tumor necrosis factor alpha (TNF-alpha). The anti-inflammatory marker included was interleukin-10 (IL-10). We adopted the criterion of extracting all data offered between groups in post-exercise recovery.

Heterogeneity was calculated via the I² statistic, where a number >50% was considered to indicate substantial heterogeneity between the tests [23]. For the “95% CI” and “Test for overall effect size” values, significant differences were assumed for *p* < 0.05. We enforced a random-effect model since this is a more conservative method that permits the study heterogeneity to fluctuate beyond chance, providing further generalizable results [17]. All data was created using the Review Manager Program (RevMan 5.4.1).

## 3. Results

### 3.1. Description of Studies

**Figure 1:**
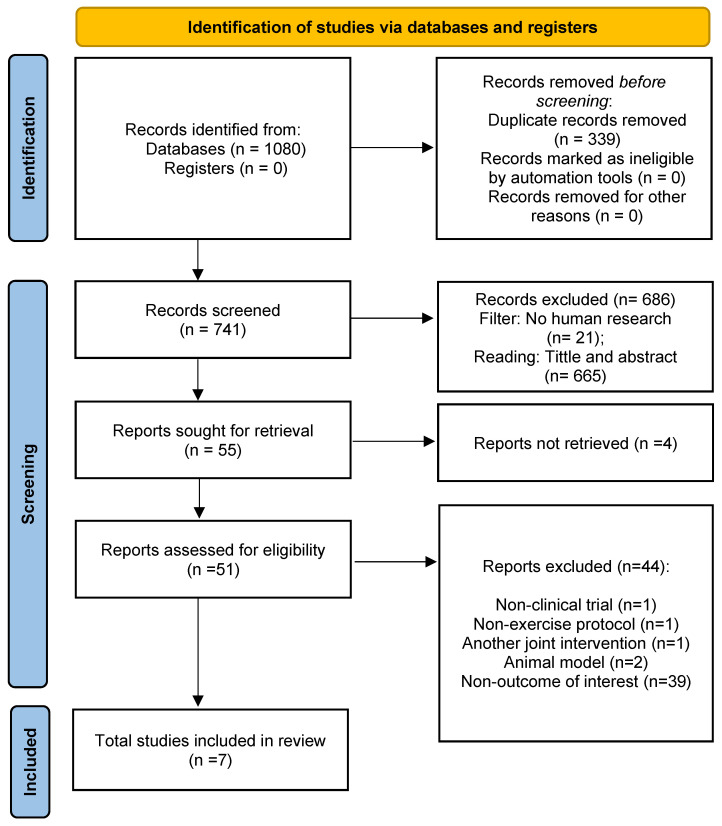
PRISMA 2020 flow diagram.

A total of 1080 studies were identified via searches in the databases. After removing duplicates (*n* = 339), 741 publications were screened for inclusion. Amongst them, 686 records were excluded after reviewing the title/abstract. The remaining 55 papers were designated for full-text reading. Lastly, seven studies [12,13,14,16,17,18,19] were included in the qualitative analysis (systematic review). Characteristics of studies included are provided in Table 1. All studies were involved in quantitative synthesis (meta-analysis). Amongst them, four studies assessed oxidative stress [14,16,17,18], two analyzed antioxidants [14,19], and three analyzed inflammatory and anti-inflammatory markers [12,13,14]. The search process and selection step details are established in the PRISMA protocol flow diagram (see Figure 1).

The studies included in this review were published between 2009 and 2022 (Table 1). Five studies were finalized in Brazil [12,13,14,16,17], one in Spain [18] and one in Iran [19]. Five references used RCT (crossover) study design [12,13,14,17,19] and two studies used RCT (parallel) study design [16,18].

Most of the assessed studies in our sample were applied to physically inactive individuals. Only three articles required physically active individuals [16,18,19]. The references exhibited similarities in visit intervals (washout) between the protocols. Only one study performed the tests on the same day [18], and one study was performed within two days [17]. Four studies enforced seven days in their protocols [12,13,16,19]. One study enforced an interval between three to seven days in its protocol. Sureda et al., (2009) [18] conducted the experiments of the experimental group and placebo in one day and at the same time.

Few studies analyzed were concerned with maintaining tests completed at the same time of day to standardize circadian influences. Three studies stipulated the time of day as during the morning [12,14,18] and Lima et al., 2018 stipulated the time of day as during the afternoon from 1:00 to 6:00 p.m. The other studies did not report the stipulated time of day [13,16,19].

The studies standardized the instructions before the test, with two studies recommending subjects avoid exercise and caffeine consumption before the trials [14,19]. Regarding mealtimes before the experiments, some studies were achieved with night fasting [12,13,18]. Fayh et al., (2012) [16] instructed the subjects to feed themselves for a minimum of 60 min and a maximum of 90 min prior to the session. Lima et al., 2018 [17] instructed the volunteers not to ingest arginine food sources for 24 h before each session.

The clinical trials assessed different doses of L-Arginine and L-Citrulline ingestion. The range covered 6 to 20 g. Sureda et al., (2009) [18] used 6 g, while in the study conducted by Valaei et al., (2022) [19], 12 g of L-Citrulline was used. Nascimento et al., (2017) [13] used 6 g, Fayh et al., (2012) [16] used 7 g, Lima et al., (2018) [17] used 7 g, Puga et al., (2016) [14] used 9 g and Alves et al., (2013) [12] used 20 g of L-Arginine.

Throughout the results, we needed studies that inserted more than one variable during statistical analysis because of the distinct characteristics of the groups studied within each article. For the anti-inflammatory IL-10 results, Nascimento et al., (2017) [13] evaluated two groups right after exercise: “placebo after exercise” and “intervention after exercise”. They also performed an analysis of the groups after one hour of exercise: “placebo one hour after exercise” and “intervention one hour after exercise”. Alves et al., 2013 [12] analyzed two groups one hour after exercise: “placebo one hour after exercise” and “intervention one hour after exercise”. Pulga et al., (2016) [14] performed group analyses after 45 min of exercise: “placebo 45 min after exercise” and “intervention 45 min after exercise”.

For the GPx, SOD, and catalase results, Valei et al., 2022 [19] analyzed blood samples at baseline (PRE, 15 min before exercise), immediately (IP), 10 min (10P) and 30 min (30P) after exercise in both placebo and intervention groups. Pulga et al., (2016) [14] evaluated both groups’ catalase and SOD concentration after 45 min of exercise.

Regarding inflammatory markers, Alves et al., (2013) [12] analyzed the concentration of TNF-alpha and IL-6 one hour after exercise in both groups (placebo and intervention). In Nascimento’s study, IL-6 concentrations were analyzed right after exercise in the placebo and intervention groups and one hour following exercise cessation in both groups. Pugmina et al., (2016) [14] evaluated IL-6 concentration 45 min after exercise in both groups.

Fayh et al., (2013) [16] implemented four cohorts to assess each of their outcomes (TBARS, carbonyls and uric acid): “group 1 control placebo (CP)”, “group 2 control L-Arginine (CA)”, “group 3 diabetic placebo (DP)”, and “group 4 diabetic L-Arginine (DA)”.

Lima et al., (2018) [17] analyzed plasma concentration of MDA before and after exercise in both groups (placebo and intervention groups). Puga et al., (2016) [14] evaluated MDA (μM) concentration 45 min after exercise in both groups (placebo and intervention groups). Sureda et al., (2009) [18] evaluated neutrophil MDA (mmol/106 cells), LDH (U/L), and CK (U/L) concentrators immediately (post-exercise) and 3 h after exercise (recovery) in both placebo and L-Citrulline supplemented groups.

### 3.2. Qualitative Analysis

Altogether, four studies assessed oxidative stress response after physical exercise between intervention protocols and placebo conditions. All studies revealed no significant differences between the protocols [14,16,17,18].

Antioxidant markers responses during physical exercise exhibited conflicting results between studies. One study identified a significant boost in antioxidant markers compared to the placebo group after physical exercise [19]. The other studies did not support the mentioned results. Puga et al., (2016) [14] found no significant differences between groups in postmenopausal women.

Regarding inflammatory outcomes, Alves et al., (2014) [12] confirmed a rise in IL-6 markers after exercise compared to the placebo group in HIV-Infected men. On the other hand, Nascimento et al., (2017) [13] observed attenuation of increased IL-6 levels in obese hypertensive men. Puga et al., (2016) [14] found no significant differences between groups.

Nascimento et al., (2017) [13] found a significant change in anti-inflammatory markers. The L-Arginine group demonstrated that supplementation maintained IL-10 levels immediately after exercise and 1 h later. Alves et al., (2014) [12] and Puga et al., (2016) [14] found no significant differences in these markers.

### 3.3. Analysis of the Risk of Bias

**Figure 2:**
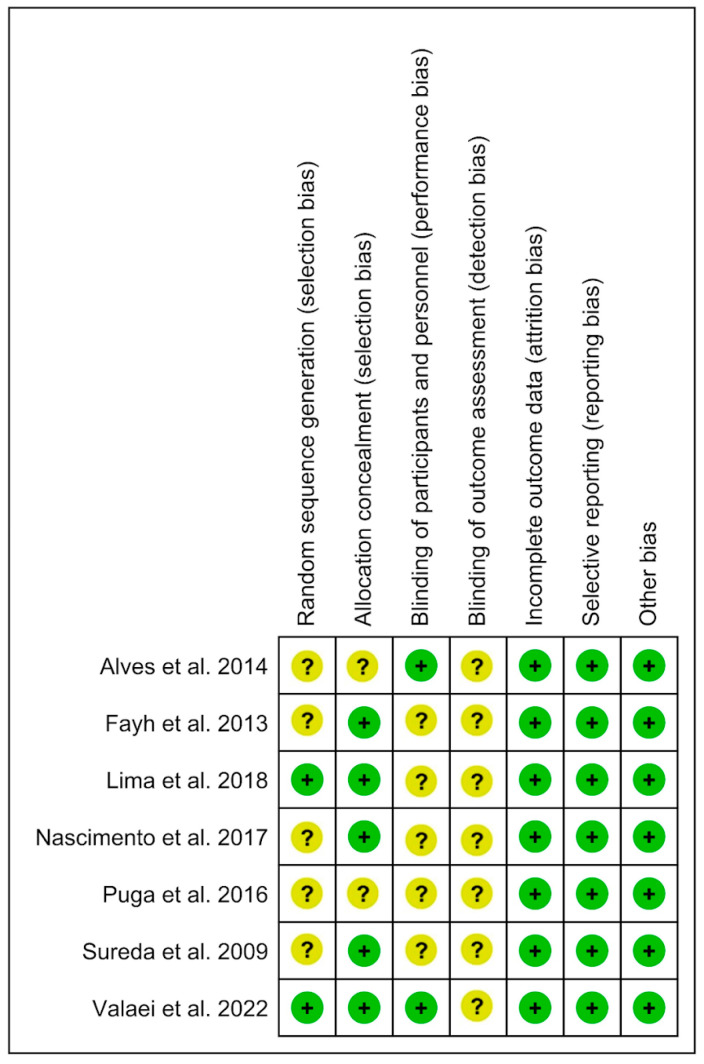
Risk of bias tool [12,13,14,16,17,18,19].

The risk of bias in the seven included studies was variable. We summarize the results in Figure 2, with further details about the risk of bias in the included studies reported in the Supplementary File “Review authors’ judgments about each risk of bias item for each included study” (Supplementary Material). Allocation: All studies (100%) enforced procedures for generating the randomization sequence, but only two provided particulars of how the process was completed. Five studies (71%) reported methods to conceal allocation. Blinding: Participant and therapist blinding and its procedures were reported in two studies (28%). Seven studies (100%) did not report blinded assessors of outcomes. Incomplete outcome data: seven references (100%) had the whole outcome data. Selective reporting: Seven studies (100%) were free of selective outcome reporting and other potential sources of bias. (See Figure 2 and “Review authors’ judgments about each risk of bias item for each included” study in Supplementary Material).

### 3.4. Quantitative Analysis

For the anti-inflammatory (IL-10), antioxidants (SOD), and inflammatory (IL-6) results, we enforced a random effect and mean difference (MD) model to quantify the effect size. In oxidative stress (MDA and TBARS) analysis, we applied a random effect and standardized mean difference (SMD) model owing to mixed indexes included in the analysis. In both analyses, the black diamond dimension represents a 95% CI. A negative effect indicates decreased values in the intervention group compared to the placebo.

### 3.5. Oxidative Stress Markers

**Figure 3:**
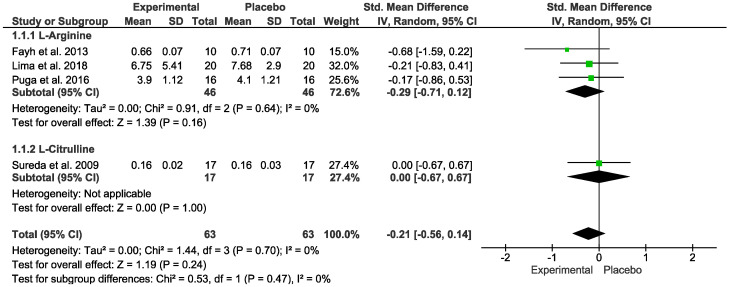
Effects of L-Arginine and L-Citrulline on oxidative stress markers [13,14,16,17,18].

No difference was detected for oxidative stress. In the “Test for overall effect”, we revealed a subtotal = −0.21 [CI: −0.56, 0.14], *p* = 0.24, and heterogeneity = 0%. In the sub-group, “L-Arginine” we found a subtotal = −0.29 [−0.71, 0.12], *p* = 0.16 and heterogeneity = 0%. For the “L-Citrulline” subgroup we observed a subtotal = 0.00 [−0.67, 0.67], *p* = 1.00, and heterogeneity was not applicable. No differences were observed between groups (*p* = 0.47), and I² = 0% (Figure 3). The GRADE quality of evidence for this result was low (Table 2).

### 3.6. Antioxidants Markers

**Figure 4:**
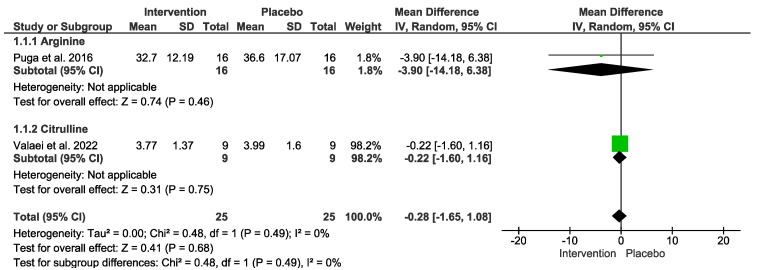
Effects of L-Arginine and L-Citrulline on antioxidant markers.

Regarding oxidants outcome, no difference was detected. In the “Test for overall effect,” we revealed a subtotal = −0.28 [−1.65, 1.08], *p* = 0.68, and heterogeneity = 0%. In the “L-Arginine” sub-group, we found a subtotal = −3.90 [−14.18, 6.38], *p* = 0.46, and heterogeneity was not applicable. For the “L-Citrulline” subgroup, we reported a subtotal = −0.22 [−1.60, 1.16], *p* = 0.75, and heterogeneity was not applicable. No differences were observed between groups (*p* = 0.49), and I² = 0% (Figure 4). The GRADE quality of evidence for this result was very low (Table 2).

### 3.7. Inflammatory Markers

**Figure 5:**
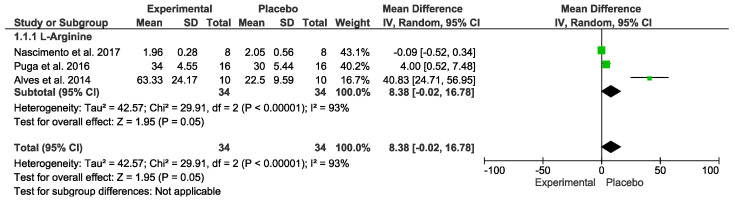
Effects of L-Arginine on inflammatory markers.

Concerning inflammatory markers, no difference was detected. In the “Test for overall effect,” we revealed a subtotal = 8.38 [−0.02, 16.78], *p* = 0.05, and heterogeneity = 93%. Testing for subgroup differences was not applicable (Figure 5). The GRADE quality of evidence for this result was very low (Table 2).

### 3.8. Anti-Inflammatory Markers

**Figure 6:**
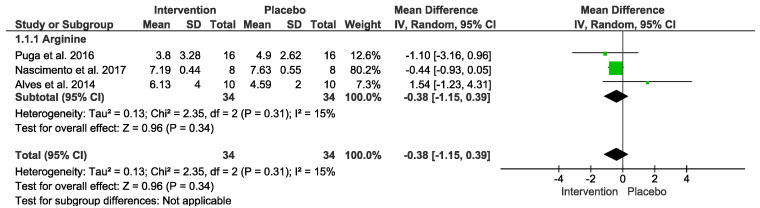
Effects of L-Arginine on anti-inflammatory markers.

No difference was detected. In the “Test for overall effect,” we revealed a subtotal = −0.38 [−1.15, 0.39], *p* = 0.34 and heterogeneity = 15%. Testing for subgroup differences was not applicable (Figure 6). The GRADE quality of evidence for this result was very low (Table 2).

We performed a sensitivity analysis to assess each study’s influence on the heterogeneity. In this analysis, the withdrawal of specific studies did not affect the total result of the meta-analysis (see sensitivity analysis in Supplementary Material). A funnel plot and meta-regression were not applied due to the small number of studies included. We consulted the Cochrane Handbook [24].

## 4. Discussion

Recent studies have evidenced that nitric oxide provides cardioprotective functions based on its role as a critical signaling messenger in vessels and the heart. This molecule has vasodilation effects and plays additional roles in the cardiovascular system [25]. Furthermore, previous data support an indirect antioxidant and anti-inflammatory influence of nitric oxide positively impacting cardiovascular disease [26]. Based on this mechanism, this systematic review and meta-analysis was undertaken to evaluate the influence of L-Citrulline and L-Arginine on inflammatory biomarkers and oxidative stress following exercise. Considering nitric oxide production depends on arginine and citrulline, we believed both supplements would improve antioxidant and anti-inflammatory markers after exercise. Nevertheless, as the main findings, we observed that (1) L-Arginine and L-Citrulline did not influence oxidative stress following effort, (2) inflammatory markers were not changed by citrulline and arginine after exercise, and (3) the meta-analysis evidenced substantial heterogeneity for oxidative stress and inflammatory and anti-inflammatory biomarkers and low heterogeneity for antioxidant biomarkers.

Exercise acutely induces metabolic changes related to oxidative stress increase [27]. This is due to high oxygen demand by active muscles during exercise, leading to higher oxygen consumption, mitochondrial activity, and presence of oxygen species [28]. The mentioned mechanism is characterized by increased pro-oxidant and reduced antioxidant activity [28]; consequently, muscle oxidative stress may lead to microlesions and muscle damage [29]. However, after repeated exercise sessions, the immune system is adapted to interact with physiological stress and in chronic exercise (e.g., athletes and well-trained subjects), the anti-oxidant responses are improved [9].

Some authors observed that L-Citrulline ingestion before exercise significantly improved antioxidant markers during recovery from effort. This is in line with the study by Forstermann and Li, (2011) [30], which reported that L-Citrulline antioxidant properties were observed in both NO-dependent and NO-independent ways. On the other hand, the study by Sureda and coworkers, (2009) [18] did not support the abovementioned data. The authors noted no changes in neutrophil MDA following exercise when the subjects ingested L-Citrulline before the exercise protocols.

Some methodological points may explain the difference between the two studies. In the study by Valaei et al., (2022) [19], the volunteers ingested 12 g of L-Citrulline powder dissolved in 200 mL of water. In contrast, the subjects from the Sureda et al., (2009) [18] study ingested 6 g of citrulline–malate dissolved in lemon juice. The volunteers from the Valaei et al., (2022) [19] study were trained young men with 2.5 + 0.4 years of experience, while the subjects evaluated in the Sureda et al., (2009) [18] study were male pre-professional cyclists. Moreover, the exercise style was different between the studies. Valaei et al., (2022) [19] evaluated volunteers subjected to high-intensity interval exercise, whereas Sureda [18] analyzed volunteers subjected to 137.1 km in 179 min of cycling exercise. In this context, our quantitative review does not provide strong data to support any benefits of L-Citrulline on post-exercise oxidative stress.

Concerning the effects of L-Arginine supplements on oxidative stress following exercise, our meta-analysis did not support any influence of this compound. The references evaluated SOD [14] for antioxidant activities while pro-oxidant status was investigated through MDA [14,17] and TBARS [16]. The three references [12,14,17] did not find a significant effect of L-Arginine on oxidative stress biomarkers after exercise. However, we should highlight that we have insufficient evidence related to this mechanism, and further studies are necessary to understand better whether or not L-Arginine influences oxidative stress following exercise.

At least one reference found positive post-exercise antioxidant effects of L-Citrulline, while all studies with L-Arginine did not find significant antioxidant influence, suggesting that L-Citrulline may present post-exercise antioxidant properties. The experimental study performed by Agarwal et al., (2017) [31] may help us to better understand this conflicting data. Based on the fact that L-Citrulline does not go through first-pass metabolism in the gut or liver and that L-Citrulline is transformed to L-Arginine in vivo [32], Agarwal et al., (2017) [31] hypothesized that L-Citrulline supplements are better than an L-Arginine diet when evaluating systemic L-Arginine. The authors studied mice separated into two groups: L-Arginine and dietary L-Citrulline treatment. They observed that L-Citrulline escaped first-pass metabolism, while 70% of the L-Arginine supplement was gone before reaching systemic circulation. The study evidenced that a L-Citrulline diet resulted in higher L-Arginine flux and a more intense increase in plasma L-Arginine levels compared to L-Arginine supplementation. It suggests that L-Citrulline supplementation is better for increasing L-Arginine content than L-Arginine supplements.

In this scenario, the metabolic changes induced by acute exercise also include inflammatory alterations. During acute exercise, serum inflammatory and anti-inflammatory biomarkers values change [33]. This is because muscles produce myokines that induce several physiological effects via paracrine or autocrine secretion during exercise [34]. When we performed the quantitative review analysis to understand the influence of arginine on inflammatory and anti-inflammatory markers following exercise, no significant influence of this intervention was reported.

The inflammatory and anti-inflammatory biomarkers used in the references evaluated by us were IL-6 [12,13,14] and IL-10 [12,13,14]. No significant differences were found between L-Citrulline vs. L-Arginine supplementation; both interventions presented no effect on inflammatory and anti-inflammatory parameters following exercise recovery.

In this context, the dose of L-Arginine and L-Citrulline is an important factor to comprehend the results in this review. As mentioned above, the study by Valaei et al. (2022) [19] evidenced significant antioxidant influence of L-Citrulline with a dosage two times higher than the dose evaluated by Sureda and coworkers, (2009) [18], which reported no significant antioxidant impact of L-Citrulline. On the other hand, the other studies failed to report antioxidant and anti-inflammatory effects of arginine following exercise recovery from analysis of administration of 6 g for seven days [13], 7 g for seven days [16], 7 g in a single dose [17], 9 g in a single dose [14], and 20 g in a single dose [12]. In this scenario, the Nascimento et al., (2017) [13] study had the highest impact on all the heterogeneity according to our sensitivity analyses (Supplementary Files). According to the authors’ results, L-Arginine was able to attenuate IL-6 increase after an exercise session in obese subjects. This is possibly because this population is susceptible to the presence of pro-inflammatory cytokines. To clarify this point and confirm whether or not L-Arginine influences IL-6 following exercise, additional clinical trials are needed in this specific population. We also hypothesize the possibility that this study is an outlier. Furthermore, our meta-analysis evidenced a high difference between results regarding oxidative stress biomarkers (heterogeneity > 50%), and not even our sensitivity analysis was not able to identify its causes. With this in mind, we suggest further primary studies with different doses of L-Arginine and L-Citrulline on different subjects to support or refute its antioxidant and anti-inflammatory properties following exercise.

Some points are worth mentioning to clarify the differences between the references evaluated. The study populations were distinct among the studies. Our systematic review included professional cyclists [18], type 1 diabetic patients [16], hypertensive volunteers [17], HIV patients [12], overweight men [13], postmenopausal women [14], and trained young healthy men [19]. Therefore, our results should not be extrapolated to people with different diseases and physical activity levels. Furthermore, we should consider these different clinical profiles as a limitation of this systematic review, given that immunological responses may differ in response to nutritional supplementation such as L-Arginine and L-Citrulline.

The exercise intensity was also different among the studies. Valaei et al., (2022) [19] submitted volunteers to an acute high-intensity interval exercise, while in the Puga et al. study (2016) [14], the subjects performed 30 min of exercise on a treadmill at the maximal lactate steady state intensity. In Nascimento’s study (2017) [13], the participants performed an acute resistance exercise, and subjects in the study by Alves (2014) [12] performed cycling for 5 min at 20 W with an increased 20 W work rate per min until fatigue; in the study by Lima et al., (2018) [17] volunteers were submitted to 60 min treadmill exercise (60−80% maximum heart rate), in the Fayh (2013) [16] study the subjects performed submaximal exercise, and in the study by Sureda (2009) [18] the participants performed cycling for 179 min. In this sense, we should also be careful when extrapolating our data to different exercise styles.

The studies included in our systematic review tried to control food intake before experiments to avoid biases in analysis. Almost all used the fasted-night state, and others gave participants recommendations about food intake before experiments. We cannot affirm the magnitude of difference that the meals before could have generated on immune responses, but we know these characteristics are essential to equivocal results.

Despite the low confidence in the results, according to the GRADE evaluation system, our study provides directions for the use of L-Arginine and L-Citrulline focused on immunological responses. Based on the diversity and heterogeneity of our results, we do not have strong data to assume that these substances may influence the immune system. Apparently, the difference in results come from different populations and doses. As we reported divergent results across primary clinical trials, we point out an important gap in the literature and now take the opportunity to suggest future studies with doses above 12 g of L-Citrulline and L-Arginine in populations more vulnerable to the production of pro-inflammatory cytokines (e.g., subjects with obesity) to investigate whether there is a relationship between the endogenous production of low nitric oxide and the physiological use of exogenously ingesting these substances.

## 5. Conclusions

This systematic review and meta-analysis indicate that L-Arginine and L-Citrulline intake before exercise has no significant effect on oxidative stress and inflammatory biomarkers following exercise.

## Figures and Tables

**Table 1:** Description of the characteristics of the study population of articles by author and year, sample, age (years), weight (kg), height (cm), body fat (%), (mean ± SD or, [min-max]), exercise protocol, average peak oxygen (mL/kg/min).

Author/ Years	Study Design	Sample	Age (Years)	Weight (kg)	Height(cm)	Body Fat (%)	Exercise Protocol	Average Peak Oxygen (mL/kg/min)	Intervention	Placebo
Alves et al., (2014) [12]	RCT (crossover)	10 HIV-1-infected men on HAART	47 ± 2	71 ± 4.7	1.68 ± 0.03	Not reported	Maximal incremental cardiopulmonary exercise tests	33 ± 8.8 (Arg) 33 ± 7.7 (Pla)	20 g of L-arg diluted in 200 mL of water	Not reported
Fayh et al., 2013 [16]	RCT (parallel)	10 young adult male subjects with uncomplicated type 1 diabetes and 20 matched control volunteers	Type I diabetics: 23.3 ± 1.73 Controls: 23.4 ± 0.59	Type I diabetics: 72.3 ± 4.25 Controls: 75.2 ± 2.54	Type I diabetics: 173.7 ± 2.18 Controls: 177.6 ± 1.81	Type I diabetics: 19.06 ± 2.92 Controls: 17.8 ± 1.20	Cycle ergometer test (during 45 min)	Type I diabetics: 37.1 ± 2.28 Controls: 45.4 ± 1.75	L-Arginine supplementation consisted of oral ingestion of identical pills containing 7 g of L-Arginine-hydrochloride	Oral ingestion of identical pills containing either amide compound
Lima et al., 2018 [17]	RCT (crossover)	20 diagnosed hypertensive patients	51.47 ± 1.24	Not reported	Not reported	Not reported	Treadmill exercise for 60 min (60–85% HRmax)	Not reported	Seven grams of lemon-flavor L-Arginine diluted in 100 mL of water	Seven grams of lemon-flavor placebo diluted in 100 mL of water
Puga et al., 2016 [14]	RCT (crossover)	16 normotensive postmenopausal women	57 ± 24	Not reported	Not reported	Not reported	Treadmill for 30 min (maximal lactate steady state)	Not reported	9 g of L-Arginine base (acid (2S)-2-amino-5-guanidopentanoic—Ajinomoto, Japan)	Placebo pill intake
Nascimento et al., 2017 [13]	RCT (crossover)	8 obese hypertensive men	46 ± 6	92.56 ± 9.9	171 ± 0.6	28.65 ± 8.58	Resistance exercise session (intensity equal to 60% of 1 repetition maximum)	Not reported	Supplements of L-arg (Sigma^®^ (Kanagawa, Japan)) gelatin capsules (6 g/day, 3 times per day and 2 g each time) were administered orally for one week	Placebo (starch) gelatin capsules (6 g/day, 3 times per day and 2 g each time) were administered orally for one week
Sureda et al., 2009 [18]	RCT (parallel)	17 volunteer male pre-professional cyclists	22.3 ± 3.71	70.6 ± 5.36	Not reported	Not reported	The cycling stage was 137.1 km long (179 min)	81.9 ± 10.72	6 g of citrulline–malate dissolved in lemon juice	The control group consumed the lemon juice vehicle alone
Valaei et al., 2022 [19]	RCT (crossover)	9 trained young men	21.41 ± 1.13	79.50 ± 9.35	183.44 ± 7.60	10.67 ± 1.52	10 min warm-up, which consisted of 5 min cycling (with the minimal workload, 50–60% of HRmax) and 5 min dynamic stretching (total body) exercise. After, participants performed twelve consecutive rounds of two-hand kettlebell swing exercise including (30 s of exercise and 30 s of rest) using a 16 kg kettlebell	Not reported	One hour before exercise, the participants consumed 12 g of L-Cit powder dissolved in 200 mL of water (L-Cit 1200 mg, NOW^®^ Sports (Arden Hills, MN, USA))	Placebo 12 g of maltodextrin with the same appearance, taste, smell and color

**Table 2:** Summary of findings (GRADE assessment): L-Arginine and L-Citrulline vs. Placebo Protocol (After Exercise) Repercussions on Oxidative Stress, Antioxidants, Inflammatory and Anti-Inflammatory Markers. Patient or population: Athletes, physically active, obese, and hypertensive subjects. HIV infected men and postmenopausal women. Intervention: Arginine or Citrulline. Comparison: Placebo.

Outcome No. of Participants (Studies)	Anticipated Absolute Effects (95% CI)		Certainty	What Happens
	Comparison	Intervention (Difference)		
Arginine or Citrulline vs. placebo after exercise (Oxidative Stress) № of participants: 63 (4 studies)	The Hedges’ g mean was 3.16	SMD −0.21 [−0.56, 0.14]	⊕⊕◯◯ LOW Due to serious risk of bias. Due to serious inconsistency. Due to strongly suspected publication bias. Upgraded because all plausible confounding would suggest spurious effect.	Intervention protocol before exercise presents no significant difference in oxidative stress index after exercise session.
Arginine or Citrulline vs. placebo after exercise (Antioxidants) № of participants: 25 (2 studies)	SOD mean was 20.29	MD −0.28 [−1.65, 1.08]	⊕◯◯◯ VERY LOW Due to serious risk of bias. Due to serious inconsistency. Due to serious imprecision. Due to strongly suspected publication bias. Upgraded because all plausible confounding would suggest spurious effect.	Intervention protocol before exercise presents no significant difference in antioxidants index after exercise session.
Arginine vs. placebo after exercise (Inflammatory) № of participants: 34 (3 studies)	IL−6 mean was 18.18	MD 8.38 [−0.02, 16.78]	⊕◯◯◯ VERY LOW Due to serious risk of bias. Due to serious inconsistency. Due to serious imprecision. Due to strongly suspected publication bias. Upgraded because all plausible confounding would suggest spurious effect.	Intervention protocol before exercise presents no significant difference in inflammatory index after exercise session.
Arginine vs. placebo after exercise (Anti-inflammatory) № of participants: 34 (3 studies)	IL−10 mean was 1.72	MD −0.38 [−1.15, 0.39]	⊕◯◯◯ VERY LOW Due to serious risk of bias. Due to serious inconsistency. Due to serious imprecision. Due to strongly suspected publication bias. Upgraded because all plausible confounding would suggest spurious effect.	Intervention protocol before exercise presents no significant difference in anti-inflammatory index after exercise session.

The risk in the intervention group (and its 95% CI) is based on the assumed risk in the comparison group and the relative effect of the intervention (and its 95% CI). CI: confidence interval; MD: mean difference; SMD: standardized mean difference; “⊕” and “◯” characterizes the evidence level.

## Data Availability

All data are provided in the journal as Supplementary Material, but additional requirements can be made through contact to authors.

## Supplementary Materials

The following supporting information can be downloaded at: https://www.mdpi.com/2072-6643/15/8/1995/s1. Supplementary File: Sensitivity analysis.
