# Supplementary material for: Absence of Effects of L-Arginine and L-Citrulline on Inflammatory Biomarkers and Oxidative Stress in Response to Physical Exercise: A Systematic Review with Meta-Analysis
Source: Nutrients. 2023 Apr 21;15(8):1995. doi: 10.3390/nu15081995 (PMC10228635; doi:10.3390/nu15081995)
Supplement: Supplementary file 1 [file nutrients-15-01995-s001.zip › nutrients-2225451-supplementary.pdf]

SENSITIVITY ANALYSIS

1. **Meta-analyzes performed without the cited study in outcome "Inflammatory"**

Authors	**Total CI**	**Total Heterogeneity**
Nascimento et al. 2017	21.54 [-14.51, 57.59]	95%
Alves et al. 2014	19.54 [-20.52, 59.61]	96%
Alves et al. 2014	1.58 [-2.36, 5.52]	81%

2. **Meta-analyzes performed without the cited study in outcome "*Anti-inflammatory*"**

Authors	**Total CI**	**Total Heterogeneity**
Puga et al. 2016	0.06 [-1.63, 1.75]	47%
Nascimento et al. 2017	0.05 [-2.52, 2.62]	56%
Alves et al. 2014	-0.48 [-0.95, -0.00]	0%
